# Network pharmacology study of the mechanism underlying the therapeutic effect of Zhujing pill and its main component oleanolic acid against diabetic retinopathy

**DOI:** 10.1042/BSR20220893

**Published:** 2023-01-30

**Authors:** Jialin Cui, Enze Shi, Yingjie Wang, Tiantian Liu

**Affiliations:** 1 School of Traditional Chinese Medicine, Capital Medical University, Beijing 100069, China; 2 Capital Medical University Affiliated Beijing Traditional Chinese Medicine Hospital, Beijing 100010, China

**Keywords:** Animal Experiment, Molecular Docking, Network Pharmacology, Oleanolic acid

## Abstract

Diabetic retinopathy (DR) is the leading cause of blindness in the working population worldwide, with few effective drugs available for its treatment in the early stages. The Zhujing pill (ZJP) is well-established to enhance the early symptoms of DR, but the mechanism underlying its therapeutic effect remains unclear. In the present study, we used systems biology and multidirectional pharmacology to screen the main active ingredients of ZJP and retrieved DrugBank and Genecards databases to obtain ‘drug-disease’ common targets. Using bioinformatics analysis, we obtained the core targets, and potential mechanisms of action of ZJP and its main components for the treatment of DR. Molecular docking was used to predict the binding sites and the binding affinity of the main active ingredients to the core targets. The predicted mechanism was verified in animal experiments. We found that the main active ingredient of ZJP was oleanolic acid, and 63 common ‘drug-disease’ targets were identified. Topological analysis and cluster analysis based on the protein–protein interaction network of the Metascape database screened the core targets as PRKCA, etc. Gene Ontology (GO) and Kyoto Encyclopedia of Genes and Genomes (KEGG) enrichment analysis showed that these core targets were significantly enriched in the pro-angiogenic pathway of the VEGF signaling pathway. Molecular docking and surface plasmon resonance revealed that ZJP and its main active component, oleanolic acid had the highest binding affinity with PKC-α, the core target of the VEGF signaling pathway. Animal experiments validated that ZJP and oleanolic acid could improve DR.

## Introduction

Diabetic retinopathy (DR) refers to retinal damage caused by chronic progressive diabetes mellitus, which has an insidious onset. Current evidence suggests that DR patients are largely in the middle to late stages when detected clinically. DR is widely recognized in ophthalmology as a microvascular complication of diabetes mellitus [1]. However, in terms of the entire retinal tissue structure, neuronal-glial cells account for approximately 95–97% (the remainder are neurovascular units), and their pathological damage is important during the pathogenesis of DR [2,3]. Hence, in DR, microangiopathy coexists with neuronal lesions, and treating microvascular damage alone is often insufficient [4]. It is well known that the main feature of diabetes is hyperglycemia (disorders of glucose metabolism) and that the risk of DR progressively increases with the duration of diabetes, yet strict glycemic control does not alter this phenomenon [5]. When the duration of diabetes exceeds 10 years, roughly 50% of patients have DR as a complication [6]. According to the International Diabetes Federation (IDF) 2021 data, about 537 million adults (20–79 years old) are affected worldwide, and China has the highest number of people with diabetes. As the global population ages, it is widely believed that this situation will become more severe. The prevalence rate for people aged 75–79 is predicted to be 24.0%, climbing to 24.7% by 2045 [7]. Therefore, the quest for effective therapeutic agents is essential to improve the outcomes of this patient population.

The effectiveness of herbal medicines in treating chronic diseases has attracted the attention of researchers worldwide [8–10]. Chinese Pharmacopeia has recorded the ZJP for ages as being an enhancing eyesight medicine, it is a combination of crude extracts from Goji Berry, Plantadodder seed, Dodder seed and other five kinds of medicinal plants.Due to the diverse composition of ZJP and the complex pathogenesis of DR, it is essential to find an effective research method.Network pharmacology integrates several disciplines (including molecular pharmacology, pharmacokinetics and bioinformatics, and network biology) and has been developed in recent years as a new and efficient approach to studying the interaction of different drugs with disease [11,12]. It allows a comprehensive integration of drugs and diseases by harnessing network analysis to systematically study the interactions between drugs, targets and diseases and to elucidate possible pre-proven pharmacological mechanisms of drugs [13–15].

**Figure 1:**
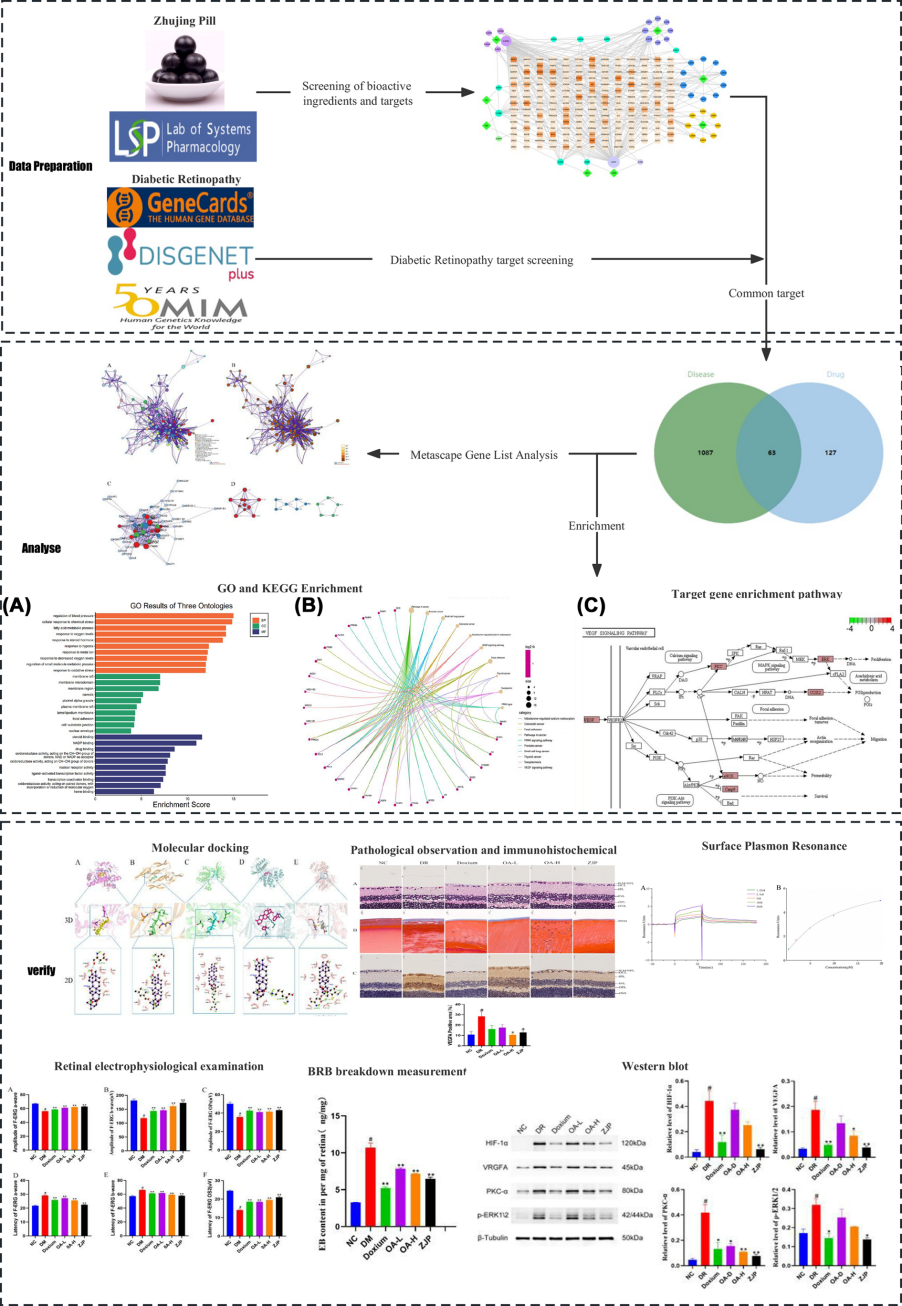
Schematic diagram of the experimental process All data were expressed as the mean ± SD (*n*=3),Compared with the normal group, #*P*<0.01; Compared With The Model Group, *P<0.05 Or **P<0.01.

In this research, network pharmacology was used to disclose the potential pharmacological mechanisms of ZJP to improve DR. Initially, multiple databases were used to screen the potential target genes of ZJP and DR. Next, the protein–protein interaction network (PPI) of the therapeutic effect of DR was constructed utilizing the metascape database, and key genes were screened using Cytoscape software. Moreover, all candidate genes underwent GO enrichment and KEGG pathway analysis. Finally, molecular docking and animal experiments were conducted to verify the molecular mechanisms and pharmacological effects of the ZJP and its main component, oleanolic acid, in DR (Figure 1).

## Materials and methods

### Screening of bioactive components and targets of ZJP

Drug ingredient screening: active ingredients of ZJP were searched in The traditional Chinese medicine systems pharmacology database and analysis platform (TCMSP) database (http://tcmspw.com/tcmsp.php) [16]. Active ingredient target screening: the SDF structures of the above ingredients were obtained using the Pubchem database (https://pubchem.ncbi.nlm.nih.gov/) [17]. The SDF structures were imported into the SwissTarget Prediction database (http://swisstargetprediction.ch/) to obtain the drug targets [18]. All targets were normalized according to the Uniprot database (https://www.uniprot.org) [19]. Using Cytoscape 8.0.0 software, a “drug-target” network diagram was created, and the active ingredients of ZJP were evaluated using Network Analyzer.

### Screening of common targets between ZJP and diabetic retinopathy and active ingredients

The GeneCards database (https://www.genecards.org/) [20], OMIM database (http://www.omim.org) [21], TTD database (http://db.idrblab.net/ttd/) [22], DRUGBANK database (https://go.drugbank.com/) [23] were retrieved using ‘Diabetic retinopathy’ as the keyword, and the Score was set ≥median value after merging and de-weighting to screen the disease targets.

The intersection of the two targets was taken, and a Venn plot was drawn using the microbiology letter online software mapping tool platform (https://www.bioinformatics.com.cn/).

### PPI network construction

Common targets were investigated using the Metascape platform (https://metascape.org/) [24] to obtain the Metascape Gene List Analysis Report. The obtained PPI networks were imported into Cytoscape 8.0.0 for optimization.

### GO enrichment analysis and KEGG enrichment analysis

Key target genes underwent GO and KEGG enrichment analyses based on R software using the Bioconductor bioinformatics package [25]. The criteria for significance included *P*-value<0.05 and *Q*-value<0.05, and the results were output as bar and bubble plots.

### Molecular docking

The crystal structures of the αA structural domains of PKC-α, ERK1/2, VEGFA, and HIF-1α used for docking were obtained from the PDB database (https://www.rcsb.org/) [26]. The 3D structures of the small molecules celastrol, tripdiolide, and triptolide, were obtained from the PubChem database, and energy minimization studies were conducted using the MMFF94 force field. In this study, molecular docking analysis was performed using AutoDock Vina 1.1.2 software (https://vina.scripps.edu/) [27], and all receptor proteins were processed using PyMol 2.5 (https://pymol.org/2/) [28] before the start of docking, including the removal of water molecules, salt ions, and small molecules. The docking box was then set up using the PyMol plugin center_of_mass.py to define the center of the docking box based on the position of the crystal ligand, with the box side length set to 30 Å. In addition, ADFRsuite 1.0 was used to convert all processed small molecules and receptor proteins into the PDBQT format necessary for AutoDock Vina 1.1.2 docking. For docking, the exhaustiveness of the global search was set to 32, and the remaining parameters were kept at their default settings. The output docked conformation with the highest score was considered the binding conformation, and the docking results were visualized and analyzed using PyMol 2.5 in combination with PLIP 2.1.7 software.

### Animal experiment

#### Model preparation and grouping

Sixty SPF grade Sprague Dawley male rats (8 weeks, weight 200–220 g) with ad libitum access to food and liquids were selected. There were housed in the Animal Experiment Center of Capital Medical University (There is no animal breeding site in this laboratory). After 1 week of adaptive feeding, the rats were randomly split into Negative control (NC, *n*=10) and DR groups (DR, *n*=50). DR group was injected by intravenous injection of STZ (60 mg/kg) (Sigma-Aldrich, St.Louis, MO, U.S.A.) for one time [29], and Type 2 diabetes was induced by high-fat diet (HFD: 10% lard, 20% sucrose, 2.5% cholesterol, 0.5% sodium chocolate and 67% basic feed). The NC group was injected with an equal volume of citrate buffer (pH 4.5) and fed a normal diet. The rats with successful modeling were randomly divided into five groups: DR group (DR, *n*=10), doxium group (doxium, *n*=10), oleanolic acid-low group (OA-L, *n*=10), oleanolic acid-high group (OA-H, *n*=10), and ZJP (ZJP, *n*=10), which were continuously fed with a high-fat diet. After 12 weeks of intragastric administration, an animal anesthesia ventilator (anesthetics: isoflurane; maintenance concentration: 2–2.5%; duration: 3–5 min) was used for anesthesia (rats were removed from the induction box, and their heads were placed in an anesthesia mask for fixation, and we ensured that the rats were fully anesthetized). The rats were anesthesized and killed by cardiac perfusion with during the retinal leakage test. The Capital Medical University’s Institutional Animal Care and Use Committee approved all animal experiments and followed the Guidelines for the Care and Use of Laboratory Animals by the National Institutes of Health [30,31] (Ethics registration number: AEEI-2019-065).

#### Administration method

The Doxium group was treated with Doxium (100 mg/kg, p.o), the OA-L group was gavaged with oleanolic acid (40 mg/kg, p.o), the OA-H group was treated with oleanolic acid (80 mg/kg, p.o), and the ZJP group was gavaged with resident pills (225 mg/kg, p.o). The NC and DR groups were gavaged with equal amounts of distilled water. All treatments were administered for 12 weeks [32–34].

#### Retinal electrophysiology

Assays were conducted at week 12 of administration. After acclimation in a dark box for 30 min, the rats were anesthetized with isoflurane, and the pupils were dilated with tropicamide eye drops. Corneal electrodes were placed at the corneas of both eyes, and grounding electrodes were casually pricked subcutaneously. The F-ERG examination was performed after dark adaptation for 5 min after penetrating the reference electrodes in the bilateral buccal mucosa with international standard white light stimulation (single stimulation, 5 s interval, 8 stimulations). The a-wave and b-wave amplitudes and peak latencies, as well as the oscillatory potentials (OPs) of F-ERG, were recorded in both eyes.

#### Blood–retinal barrier (BRB) leakage detection

Evans Blue (EB) was dissolved in saline and injected into the tail vein of deeply anesthetized rats (40 mg/kg), and the thoracic cavity was opened after 2 h of circulation. A 1% paraformaldehyde solution (pH 4.5) prepared with sodium citrate buffer (37°C) was perfused into the left ventricle for 2 min. Immediately after perfusion, rat eyes were removed, retinas were separated, vacuum dried for 5 h and weighed on an electronic balance. The individual retinas were extracted in 120 μl formamide in a water bath (70°C) for 18 h and then transferred to ultrafiltration centrifuge tubes. About 50 μl of the supernatant was extracted by centrifugation at 12 000 r/min for 45 min at 4°C. About 120 μl of formamide was added to each tube to extract Evans blue from the atrial water and retinas; 100 μl of the supernatant was extracted by centrifugation and measured by a light spectrophotometer. The concentration of Evans blue was measured by spectrophotometer (Evans blue absorption peak 620 nm, absorption trough 740 nm, OD = OD_620–740 nm_). Moreover, the concentration of Evans blue in the supernatant was calculated by standard curve analysis.

#### Histopathological observation of the retina

Paraffin-embedded tissue sections with a thickness of 5 μm were taken and stained with hematoxylin–eosin (HE) to observe the retinal tissue structure using a light microscope; another tissue section was treated with Masson staining to observe the inner boundary membrane tissue morphology.

#### Immunohistochemistry of VEGF in retinal tissue

Paraffin-embedded tissue sections with a thickness of 5 μm were taken for routine dewaxing, antigen repair and staining (the sections must be kept in a wet state at any time during the production process). After sealing the sections, observation was performed under a microscope and results were statistically analyzed.

#### Western blot was used to detect pathway and protein expression changes

The total protein was extracted from each group of rat retinal tissues, quantified by the BCA method, and 30 μg per group was taken by SDS-PAGE gel electrophoresis, transferred to PVDF membrane after electrophoresis was completed, and closed in skimmed milk for 1 h. The tissues were incubated in primary antibody diluted at 1:1000 overnight, washed 3 times with TBST, and incubated in secondary antibody diluted at 1:10 000 for 1 h at room temperature, and HIF-1α, VEGFA, PKC-α, and ERK1/2 protein expression were detected in each group, and β-Tubulin was used as the internal reference.

### SPR analysis of the binding ability of drug small molecules and proteins

#### Protein microarray fixation

When the temperature of the computer and SPR system to be tested reached the preset temperature of 25°C, the SPR control software was started and connected to the host system. Then, according to the isoelectric point of PKC-α protein, the optimal pH value and concentration range of protein fixed on the chip were screened to ensure that more protein was fixed on the protein chip.

#### Oleanolic acid test solution preparation

Oleanolic acid was modified with polyethylene glycol (*M*_r_ = 5000) to improve its solubility and availability at preset temperatures. PEG-OA was accurately weighed and then prepared into 20 nM small molecule stock solution with PBS solution, diluted into 10, 5, 2.5 and 1.25 nM reference solution with mobile phase solution.

#### Affinity detection

The LMW kinetics/affinity multi-cycle module of SPR system software was used; the binding and dissociation times were set to 60 and 180 s, respectively. After dissolution correction, the response signals recorded during protein binding and dissociation from the small-molecule drug were fitted to the data results using Biacore S200 Evaluation Software.

### Statistical method

SPSS 20.0 statistical software was used for data analysis, and data were expressed as ‘mean ± standard deviation’ (*x* ± s). The differences between groups were analyzed by one-way ANOVA, and a *P*-value < 0.05 was statistically significant.

## Results

### Screening of putative bioactive compounds and targets of ZJP

A detailed composition of ZJP is shown in Table 1. The active ingredients of ZJP were screened in the TCMSP database using the parameters oral-bioavailability (OB) ≥ 30%, drug-likeness (DL) ≥ 0.18. A total of 42 main active ingredients were obtained.

**Table 1:** Composition of ZJP

Chinese name	Botanical name	Genus	Family	Weight (g)	Part used
Tusizi	Cuscuta chinensis Lam	Cuscuta	Convolvulaceae	24	Seed
Cheqianzi	Plantago asiatica L	Plantago	Plantaginaceae	9	Seed
Chushizi	Broussonetia papyrifera(L.)Vent	Broussonetia	Moraceae	3	Fruit
Gouqizi	Lycium barbarum L	Lycium	Solanaceae	6	Fruit
Wuweizi	*Schisandra chinensis* (Turcz.) Baill	*Schisandra* Michx	Magnoliaceae Juss	6	Fruit
Danggui	Angelica sinensis (Oliv.) Diels	Angelica L	Apiaceae Lindl	15	Root
Dihuang	Rehmannia glutinosa (Gaert.) Libosch	Rehmannia	Scrophulariaceae	15	Root
Huajiao	Zanthoxylum bungeanum Maxim	Zanthoxylum L	Rutaceae Juss	3	pericarp

**Figure 2:**
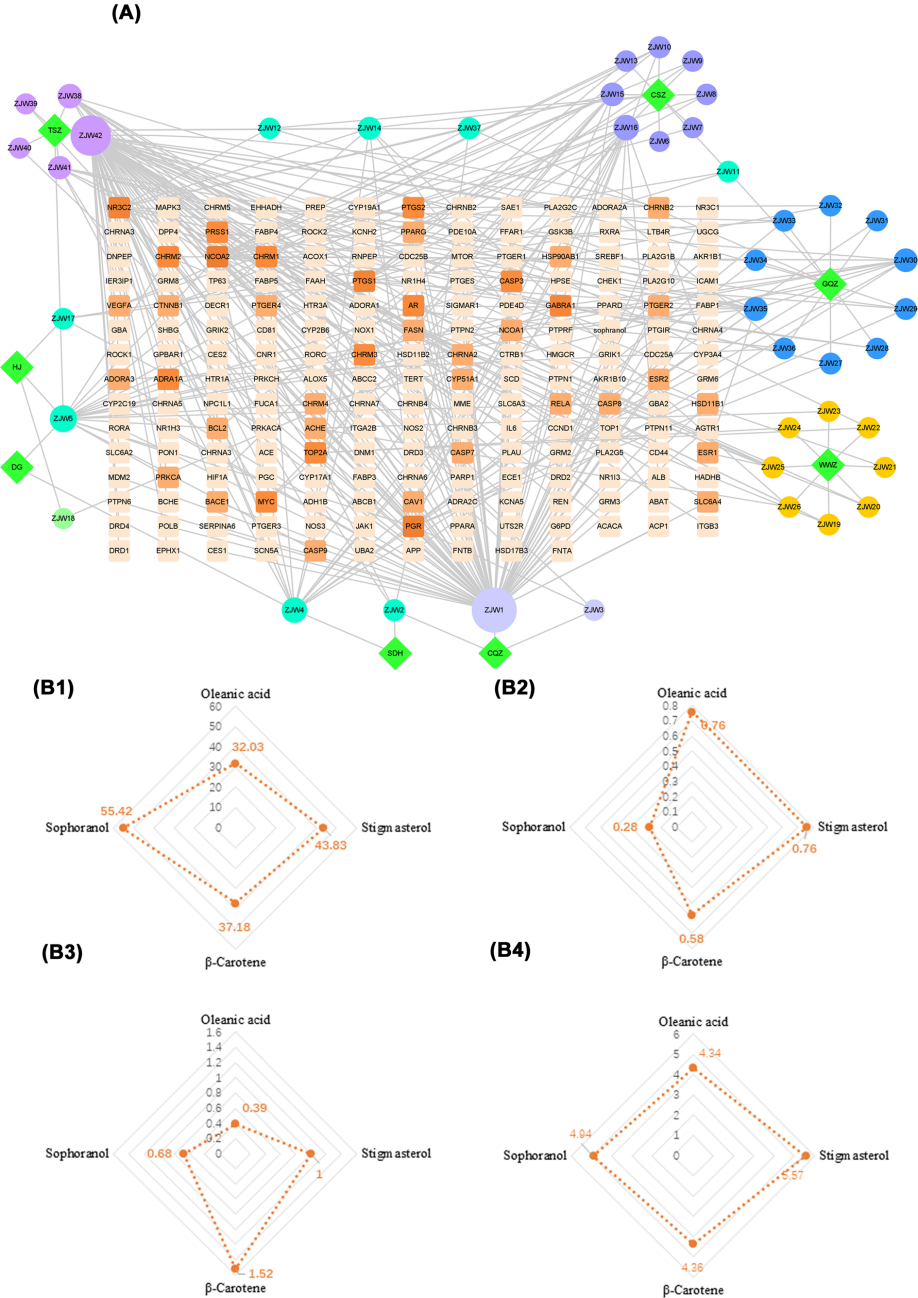
ZJP drug-disease target convergence map (**A**) Target map of effective active components of ZJP. The darker the color, the larger the node and the higher the degree. (**B1**) Oral-bioavailability. (**B2**) Drug-likeness. (**B3**) Drug Blood–brain barrier penetration. (**B4**) Drug half-life.

The SDF structures of 42 active ingredients of ZJP were obtained using the PubChem database, and 116 drug targets with scores >0.1 were obtained from the SwissTarget Prediction database. Using Cytoscape 8.0.0 software, a ‘drug-target’ network diagram was constructed, and the Network Analyzer function was used to analyze the main active ingredients of ZJP (Figure 2A). The circles represent the active ingredients of ZJP (*n*=42), and the squares are the targets (*n*=192). The area and color of the graph deepen and become larger with increasing degree value, indicating the more important the ingredient. ZJP1 (Oleanolic acid), ZJP42 (Sophranol), ZJP4 (Stigmasterol), and ZJP16 (β-carotene) had the highest degree values, especially oleanolic acid (OA) (Figure 2B).

### Screening of common drug targets of active components of ZJP and diseases

The keywords ‘diabetic retinopathy’ were searched in OMIM, Disgenet, and Genecards databases, and a target score ≥ median value was used to identify significant disease targets. After combining and de-weighting the obtained disease targets, 1150 disease targets were screened.

The intersection of the screened active ingredient targets of ZJP and its disease targets during DR treatment yielded 63 common targets, visualized in a Venn plot generated by Microscience software.

### Construction of the protein–protein interaction network

The 63 common targets were imported into the Metascape platform, and ‘homo sapiens’ was selected to analyze the common targets. We first determined all statistical enrichment terms (GO terms and KEGG pathways), calculated the cumulative hypergeometric p-value and enrichment factor, and used them for filtering. Subsequently, according to the kappa statistical similarity between gene members, the remaining essential terms were clustered into a tree.Kappa was screened with a threshold of 0.3 and plotted. Subsequently, we selected a subset of representative terms from this cluster and converted them into a network layout (Supplementary Figure S1A and B). Specifically, each term is represented by a circular node whose size is proportional to the number of input genes in the term, and its color indicates its cluster identity (i.e., nodes of the same color belong to the same cluster). Terms with a similarity score > 0.3 were connected by an edge (the width of the edge represents the similarity score). The network was visualized using Cytoscape, which adopts a ‘force-oriented’ layout with clear edge binding.

#### Core target screening based on topology analysis

The PPI network retrieved from the Metascape platform was then imported into Cytoscape 8.0.0 for optimization (Supplementary Figure S1C). The topology analysis was performed by the Network Analyzer tool, and the four parameters, degree, betweenness centrality, average shortest path length and closeness centrality, were used as reference standards to obtain genes with scores greater than the average score as core targets, including MAPK3, RELA, ESR1, PRKCA, BCL2, HIF-1α, PTGS2, IL6, VEGFA, and so on.

#### Core target screening based on cluster analysis

The constructed PPI network was imported into Cytoscape 8.0.0, and the analysis of gene clusters and the screening of core targets were executed using the MCODE module. A total of three gene clusters and three core genes were obtained, and the core genes were CTNNB1, CCND1, and HIF-1α (Supplementary Figure S1D).

### GO annotation and KEGG pathway enrichment analysis

**Figure 3:**
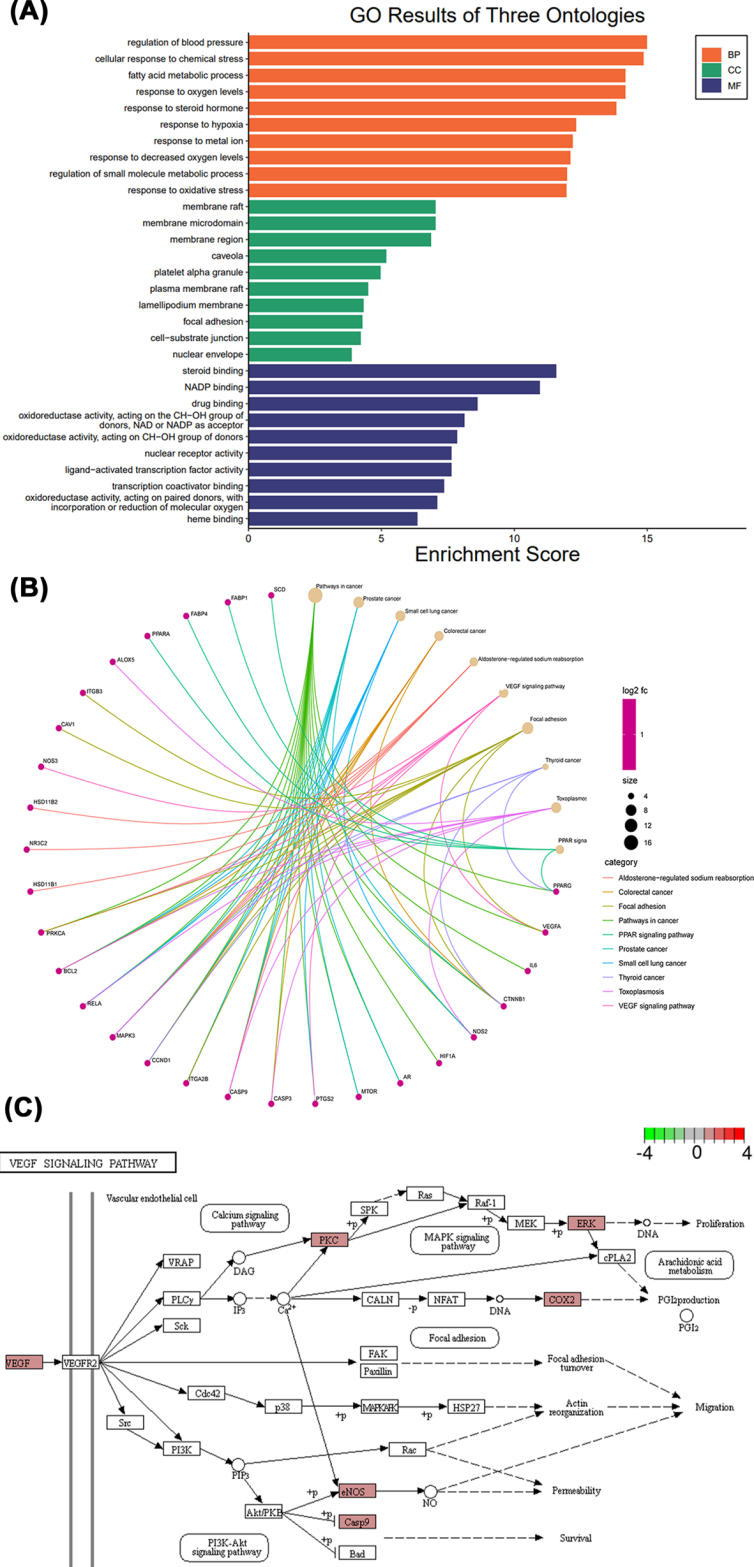
Enrichment analysis results (**A**) GO annotation results. (**B**) KEGG pathway cnetplot. (**C**) VEGF signaling pathway.

GO analysis of the 63 common targets was conducted using R language and divided into biological processes, cellular components and molecular functions (Figure 3A). GO annotation showed significant enrichment in a total of 3302 biological process pathways, including regulation of blood pressure (GO:0008217), cellular response to chemical stress (GO:0062197), fatty acid metabolic process (GO:0006631), response to oxygen levels (GO:0070482), response to steroid hormone (GO:0048545), response to hypoxia (GO:0001666), response to metal ion (GO:0010038), response to decreased oxygen levels (GO:0036293), and regulation of small molecule metabolic process (GO:0062012), and response to oxidative stress (GO:0006979). Besides, a total of 229 cellular components exhibited significant enrichment, mainly involving membrane plasma membrane raft (GO:0044853), lamellipodium membrane (GO:0031258), focal adhesion (GO:0005925), cell–substrate junction (GO:0030055), nuclear envelope (GO:0005635). Moreover, significant enrichment was observed in a total of 308 processes related to molecular function, mainly steroid binding (GO:0005496), NADP binding (GO:0050661), drug binding (GO:0008144), oxidoreductase activity (GO:0016614, GO:0016614, GO:0016705), nuclear receptor activity (GO:0004879), ligand-activated transcription factor activity (GO:0098531), transcription coactivator binding (GO:0001223), and heme binding (GO:0020037).

A total of 125 KEGG pathways were significantly enriched, and the top 10 results (Figure 3B) included pathways in cancer (hsa05200), prostate cancer (hsa05215), small cell lung cancer (hsa05222), colorectal cancer (hsa05210), aldosterone-regulated sodium reabsorption (hsa04960), VEGF signaling pathway (hsa04370), focal adhesion (hsa04510), thyroid cancer (hsa05216), toxoplasmosis (hsa05145), and PPAR signaling pathway (hsa03320). Based on our PPI network and GO annotation results, the VEGF signaling pathway was regarded as a key mechanism for ZJP and its main active ingredient, oleanolic acid, in treating DR (Figure 3C).

### Molecular docking verification

**Figure 4:**
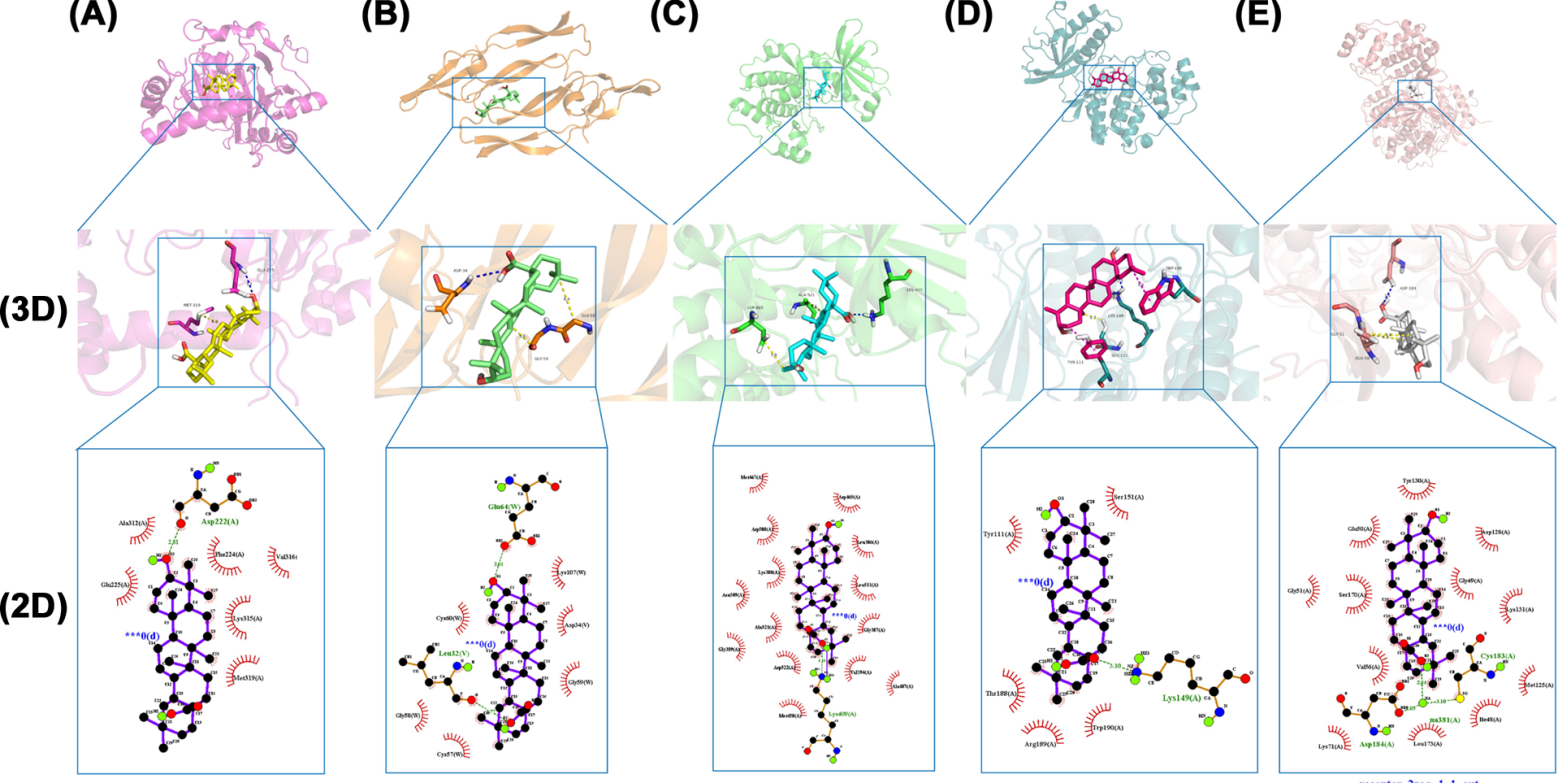
Oleanolic acid molecular docking results (**A**) HIF-1α-, (**B**) VEGFA, (**C**) PKC-α, (**D**) ERK1 and (**E**) ERK2.3D figures show the molecular model of oleanolic acid in the binding pocket of the protein. 2D figures of the interactions between Oleanolic acid and surrounding binding sites. Hydrogen bonds were displayed as green dashed lines. Hydrophobic interactions are indicated as red opposite arcs.

Molecular docking of PKC-α, ERK1/2, VEGFA, and HIF-1α, key targets enriched in the VEGF signaling pathway, with oleanolic acid was performed. It is generally accepted that an affinity of < −6 kcal mol^−1^ indicates a good binding effect, while < −9 kcal mol^−1^ indicates a very good binding effect. As seen in Table 2, oleanolic acid exhibited good affinity with PKC-α. Molecular docking by Autodock showed that the binding free energy was relatively high (Figure 4C). Overall, these results showed that oleanolic acid had a good affinity for PKC-α.

**Table 2:** Docking of oleanolic acid with predicted target molecules

Target	Hydrogen bonding	Hydrophobic interaction	π–π interaction	Binding energy (kcal mol^–1^)
HIF-1α	GLU225	MET319		−5.4
VEGFA	ASP34	GLY59 GLY58		--6.7
PKC-α	LYS409	ASP475 ALA521		−9.2
ERK1	SER151	LYS149	TRP190 TYR111	--7
ERK2	ASP184	GLY51 GLU50		−5.4

### Animal experiment results

#### Results of retinal electrophysiological examination

**Figure 5:**
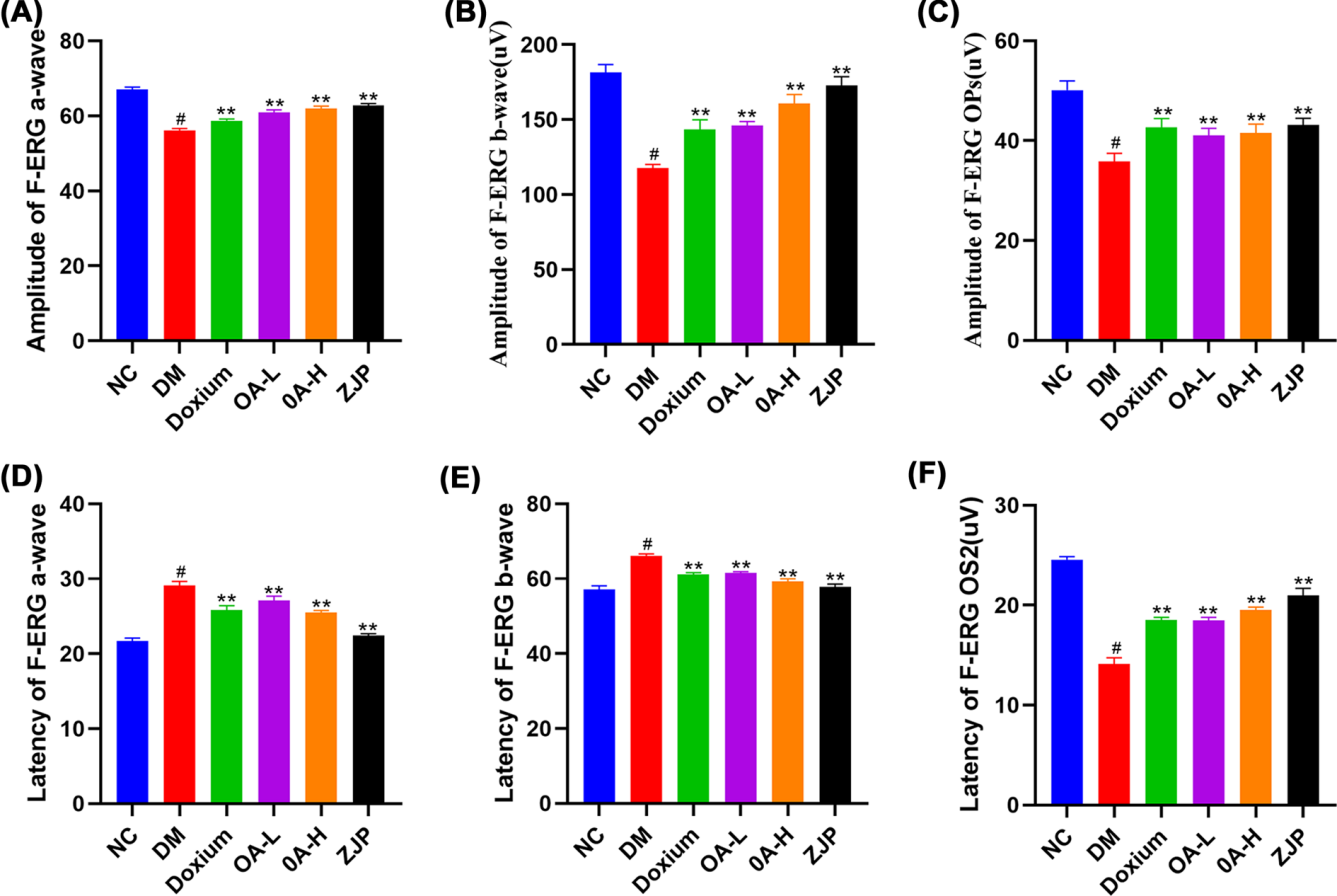
Retinal electrophysiological examination Panels (**A–C**) are the a-wave, b-wave and Ops amplitude of F-ERG;panel (**D–F**) represent the a-wave, b-wave and OS2 latency of F-ERG. All data were expressed as the mean ± SD (*n*=3). Compared with the normal group, #*P*<0.01; Compared with the model group, **P<0.01.

For F-ERG, a-wave (Figure 5A) and b-wave (Figure 5B) amplitudes were significantly lower in the DR group compared with the NC group, while the a-wave (Figure 5D) and b-wave (Figure 5E) latencies were higher in the DR group than in the NC group. Each treatment significantly increased the amplitude of a- and b-waves and normalized the a- and b-wave latencies. The DR group decreased significantly compared with the OPs and OS2 in the NC group. Amplitude increased in each group after treatment but remained lower than in the NC group (Figure 5C,F).

#### Blood–retinal barrier (BRB) breakdown measurement

**Figure 6:**
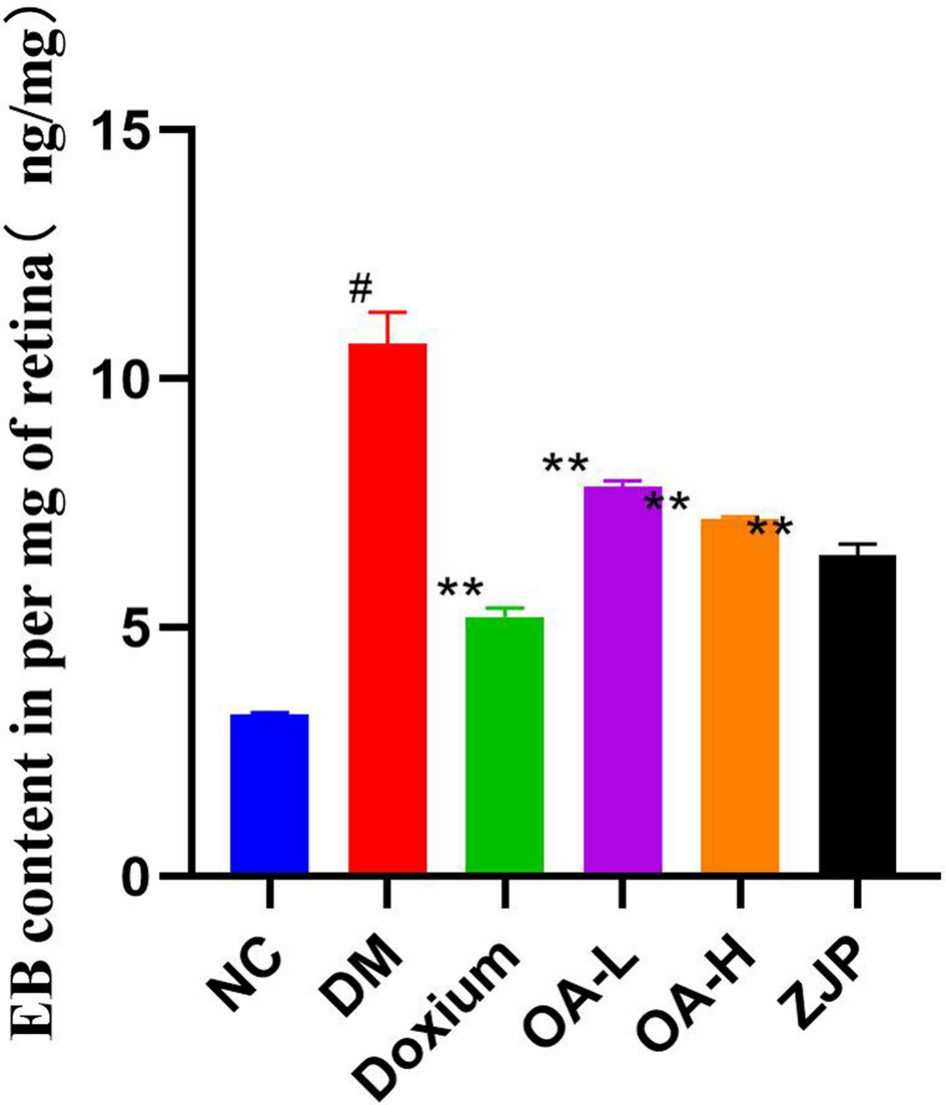
EB content of retina All data were expressed as the mean ± SD (*n*=3). Compared with the normal group, #*P*<0.01; Compared with the model group, *P<0.05 or **P<0.01.

Evans Blue leakage was significantly higher in DR compared with the NC group. Moreover, the mean leakage in each treatment group was significantly reduced compared to the model group (Figure 6).

#### Histopathological observation of the retina

**Figure 7:**
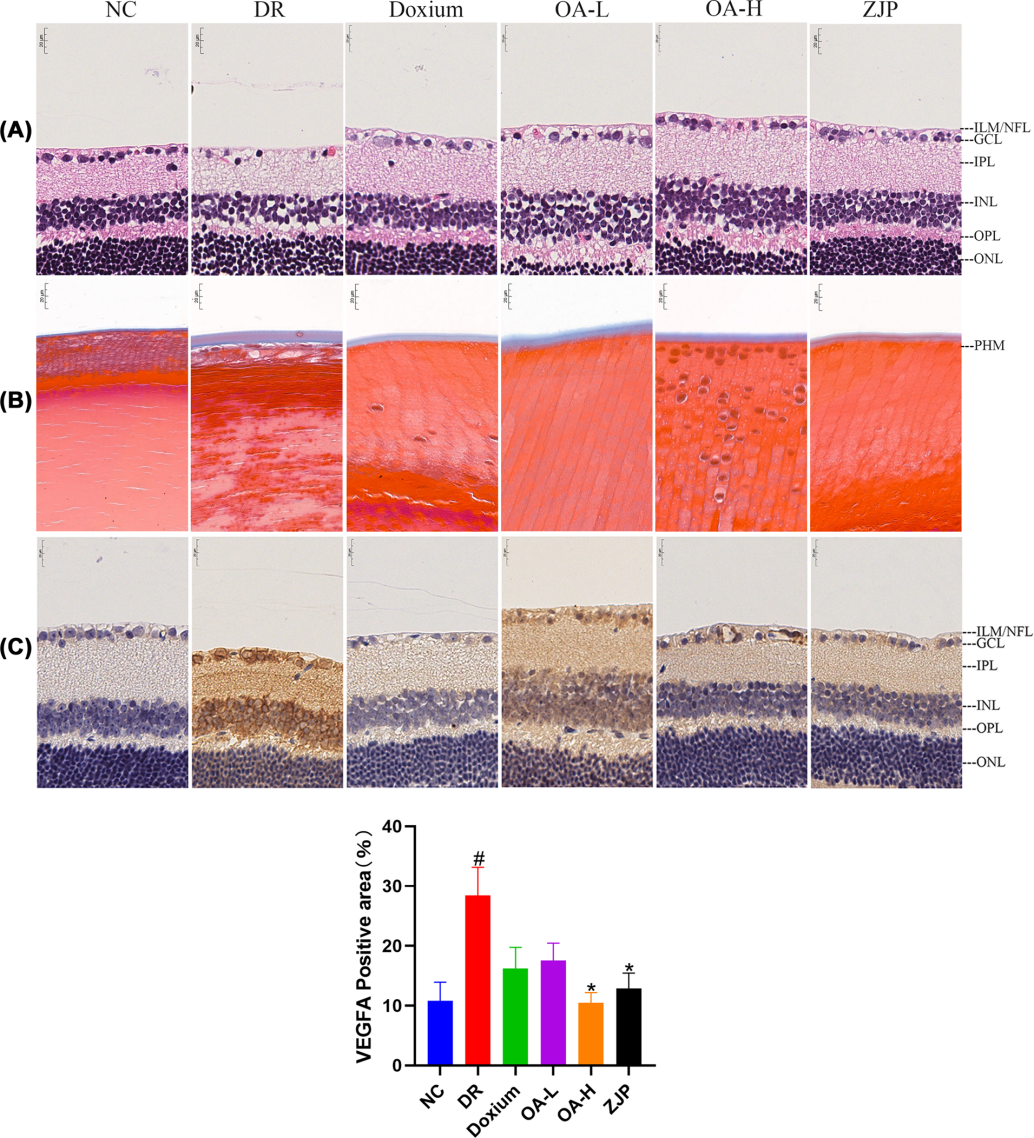
Retinal histopathological observation (**A**) HE staining × 400. (**B**) Masson staining × 400. (**C**) VEGFA immunohistochemistry × 400. All data were expressed as the mean ± SD (*n*=3). Compared with the normal group, #*P*<0.01; Compared with the model group, *P<0.05.

The NC group rats exhibited a neat arrangement of retinal layers, normal ganglion cells, no edema, and atrophy. DR rats had substantially fewer retinal ganglion cells, vacuole-like changes in cells, and fewer cells in the inner nuclear layer exhibiting atrophy. Doxium treatment group rats had reduced retinal ganglion cells, vacuole-like changes of cells, neater arrangement of the inner nuclear layer, and no edema. Retinal ganglion cells of rats in the OA-L group presented vacuolar-like changes, disorganized arrangement of the inner nuclear layer and significant edema. Retinal ganglion cells in the OA-H group exhibited vacuolar-like changes, with a neat arrangement of the inner nuclear layer and mild edema. In the ZJP group, the retinal layers were systematically organized, and the ganglion cells were vacuolated (Figure 7A).

Compared with the NC group, the collagen fibers of the posterior hyaloid membrane were considerably proliferated in DR rats. Moreover, the proliferation of collagen fibers of the posterior hyaloid membrane was reduced in each treatment group compared with the DR group (Figure 7B).

#### Immunohistochemistry of VEGFA in retinal tissue

Compared with the NC group, VEGFA expression was significantly increased in the retinal tissue of DR rats. Compared with DR, VEGFA expression in retinal tissues of rats in the Doxium group, OA-H group and ZJP group was decreased to varying degrees (Figure 7C). In this respect, VEGFA was more concentrated in the retinal nerve fiber layer.

#### Detection of VEGF signaling pathway and prediction of target protein expression by Western blot

**Figure 8:**
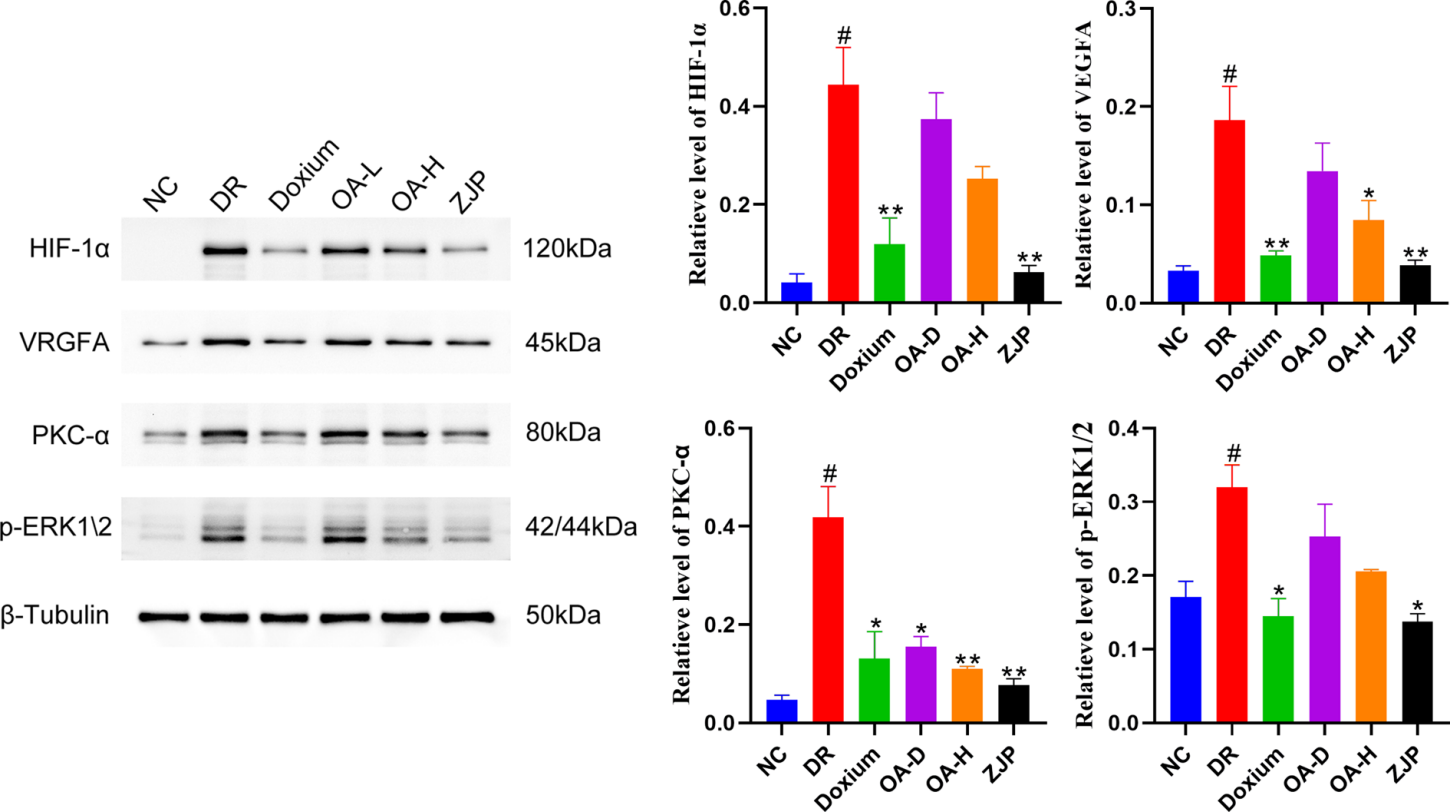
Western blot was used to detect HIF-1 α, VEGFA, PKC-α and P-ERKK1/2 expression in the retina All data were expressed as the mean ± SD (*n*=3). Compared with the normal group, #*P*<0.01; Compared with the model group, *P<0.05 or **P<0.01.

Compared with the NC group, the expression of HIF-1α, VEGFA, PKC-α, and P-ERK1/2 in the retinal tissues of DR rats was significantly higher. Moreover, the expression of HIF-1α, VEGFA, PKC-α, and P-ERKK1/2 in the retinal tissues of rats in the Doxium group, OA-H group and ZJP group exhibited different degrees of reduction (Figure 8).

### Binding capacity of PKC-α to oleanolic acid

**Figure 9:**
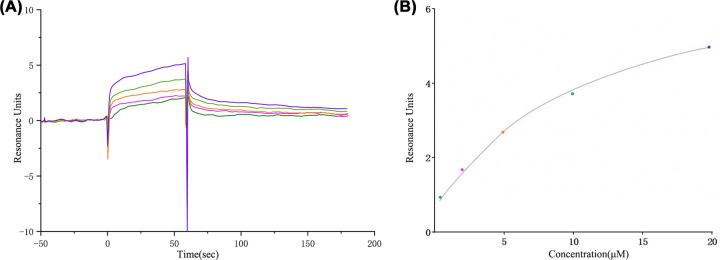
The results of the SPR experiment (**A**) The SPR titration curve of Oleanolic acid with PKC-α protein (from top to bottom, the concentration was 20, 10, 5, 2.5, and 1.25 μm). (**B**) SPR steady-state fitting diagram of the interaction between Oleanolic acid and PKC-α.

SPR binding analysis showed that the affinity constant was *K*_D_ = 1.73 μM, and oleanolic acid could be fitted to the curve and showed good affinity (Figure 9).

## Discussion

In the present study, our comprehensive database mining yielded 42 potential bioactive components, 1150 DR-related targets, and 63 common targets of ZJP and DR. PPI network analysis showed that oleanolic acid was the active ingredient with the highest nodal degree value and played a key role in ZJP to improve DR.

PRKCA, HIF-1Α, VEGFA, and MAPK were identified as significant genes based on the disease target network analysis results. It is well-established that the core of diabetic biochemical abnormalities is hyperglycemia. In hyperglycemic states, mitochondrial dysfunction causes enhanced oxidative stress and massive production of reactive oxygen species leading to altered glycolytic pathways [35], which ultimately results in increased DAG synthesis and activation of PKC-α [36–38]. The PKC-α family can cause retinal blood flow through multiple pathways, such as promoting extracellular matrix protein synthesis, leading to leukocyte adhesion and endothelial cell activation and proliferation altered kinetics and disrupting the blood–retinal barrier [39–41]. In this respect, superoxide ions and protein hydrolases can be produced when stagnant leukocytes increase in size and adhere to vascular endothelial cells [42]. At the same time, the enlarged leukocytes have poor durability, and stagnation in the vasculature can cause impaired retinal capillary microcirculation, resulting in a lack of oxygen supply due to leaky and non-perfused microvasculature in the fundus [43–45]. This retinal ischemia and hypoxia state exacerbates the inflammatory response and induces upregulation of hypoxia-inducible factor HIF-1α [46]. It has been shown that HIF-1α acts as a transcription factor that regulates the expression of multiple midstream and downstream factors [47], including VEGFA and its receptors, which are most critical for angiogenesis [48], and activated PKC-α can promote increased expression of vascular endothelial cell growth factor [49]. Indeed, the essence of retinal neovascularization is to improve the state of retinal ischemia and hypoxia, but the lack of complete tissue coverage makes this new blood vessel structurally incomplete, thinner and more brittle compared to the normally repaired vessels, which can easily leak or rupture and bleed, damaging the normal retinal tissue structure leading to impaired vision.

Functional analyses showed that ZJP and its main component, oleanolic acid, were mainly enriched in the VEGF signaling pathway, whose main role is to promote angiogenesis while preserving existing vessels. However, vascular abnormalities may occur when endothelial growth factors are much higher than anti-vascular endothelial growth factors [50]. Vascular endothelial growth factor (VEGFA) is a direct and primary stimulating vascular growth factor that plays an important role in neovascularization [51]. Various factors associated with angiogenesis directly or indirectly induce the expression of VEGFA and its receptors to contribute to enhanced retinal microvascular endothelial cell permeability, further leading to the occlusion of retinal microvessels or pro-pathological revascularization. After retinal vascular endothelial cell injury, apoptosis and loss, endothelial progenitor cells (EPC) migrate to the injury site to participate in endothelial cell repair and replace the injured vascular endothelial cells [52]. Given their potential to proliferate, migrate, and differentiate into endothelial cells, EPCs participate in the repair of injured vascular endothelial cells and physiological and pathological neovascularization [53]. When VEGFA activates phosphatidylcholine-specific phospholipase C (PLC3) by binding to VEGFA receptors specifically expressed on retinal endothelial cells, it hydrolyzes phosphatidylinositol biphosphate to produce diglycerolipids (DAG) and inositol polyphosphate (IP), where DAG activates protein kinase C (PKC) in the cytosol and anchors it to the membrane. Later, the signal is transferred into the cell via the tyrosine-protein kinase pathway, activating the MAPK signaling pathway to stimulate the proliferation and migration of vascular endothelial cells and alter the extracellular matrix, which then induces endothelial cell growth, aggravates vascular permeability or promotes retinal neovascularization [54].

The molecular docking simulation technique is a convenient and effective way to investigate the interaction of small molecules with target targets. In the present study, we used Vina 1.1.2 software to perform docking studies of the compound Oleanolic acid with H1F-1α, VEGFA, PKC-α, P-ERK1, and P-ERK2, respectively. Our findings suggested that the PKC-α target may be a key target. Next, we evaluated the binding mode of Oleanolic acid and PKC-α, which formed hydrophobic interactions with ASP475 and ALA521 and hydrogen bonds with LYS409 (Figure 4C) [55–57].

Electroretinography is mainly used clinically to diagnose lesions between photoreceptor cells to ganglion cells []. The a-wave is mainly derived from the receptor potential of photoreceptor cells, while the b-wave is mainly derived from the conduction of visual im-pulses by bipolar cells. Oscillatory potentials are often used to diagnose DR early [58]. The OPs wave is selectively reduced or disappears at the onset of DR, reflecting an ischemic state in the retina. In the present study, OPs wave amplitude was reduced in DR rats compared with the NC group, and OPs wave amplitude was elevated in each treatment group compared to DR. The b-wave amplitude of the electroretinogram was reduced in DR rats compared with the NC group. Compared with DR, the increase in the b-wave amplitude of the electroretinogram in each treatment group suggested that ZJP and its main component, oleanolic acid, yielded a protective effect on retinal neurological impairment in diabetic rats. Moreover, we found that the EB leakage in the DR group and each treatment group was significantly higher than in the NC group, indicating that the blood-retinal barrier was damaged, and leakage increased during the early stage of diabetes mellitus in rats. EB leakage in each treatment group was reduced compared with the DR group, suggesting that ZJP and its main component, oleanolic acid, could reduce the retinal leakage caused by hyperglycemia, thus maintaining the integrity of the diabetic blood-retinal barrier.

Other related studies found that ZJP could improve weight loss in diabetic rats and inhibit the overexpression of ICAM-1, TNF-α, and IL-1β in retinal tissue [59]. Among them, modern pharmacological studies have found that Lycium barbarum has antioxidant and anti-apoptotic effects on retinal photoreceptor cells and has neuroprotective effects [60]. There is a growing consensus that Cuscuta, Broussonetia and Cyperus have antioxidant properties beneficial in treating neurodegenerative diseases and inhibit retinal photoreceptor cell apoptosis [61–63]. Moreover, oleanolic acid and its derivatives have anti-inflammatory and hypoglycemic effects and mitigate tissue edema and ischemia–reperfusion injury [64–67]. Our experimental results substantiated that ZJP and its main component, oleanolic acid, could inhibit the proliferation of collagen fibrous tissue and improve the histopathological changes in the retina of diabetic rats.

## Conclusions

In this study, we predicted the effect and mechanism of the ZJP on DR through network pharmacology prediction analysis, molecular docking and animal experiments. Network pharmacology showed that the ZJP might improve DR through multiple targets and pathways. Oleanolic acid was found to be the main active ingredient. Molecular docking showed that oleanolic acid had the most significant potential activity, similar to PKC-α. Animal experiments substantiated that oleanolic acid in ZJP and its main components might play an important role in inhibiting the VEGF signaling pathway in DR. This study provides a theoretical basis for further research on the effect of oleanolic acid in the treatment of DR.

## Supplementary Material

Supplementary Figures S1-S2

## Data Availability

The datasets used and/or analyzed during the current study are available from the corresponding author on reasonable request.

## Abbreviations

BRB: blood–retinal barrier
DR: diabetic retinopathy
EB: Evans Blue
ERK1/2: extracellular regulated protein kinases 1/2
HFD: high-fat diet
HIF-1α: hypoxia inducible factor-1
KEGG: Kyoto Encyclopedia of Genes and Genomes
PKC-α: protein kinase C-alpha
PPI: protein–protein interaction
VEGF: vascular endothelial growth factor
ZJP: Zhujing pill
