# Supplementary material for: Network pharmacology study of the mechanism underlying the therapeutic effect of Zhujing pill and its main component oleanolic acid against diabetic retinopathy
Source: Biosci Rep. 2023 Jan 30;43(1):BSR20220893. doi: 10.1042/BSR20220893 (PMC9894013; doi:10.1042/BSR20220893)
Supplement: Supplementary Figures S1-S2 [file BSR-2022-0893_supp.pdf]

Supplementary Materials

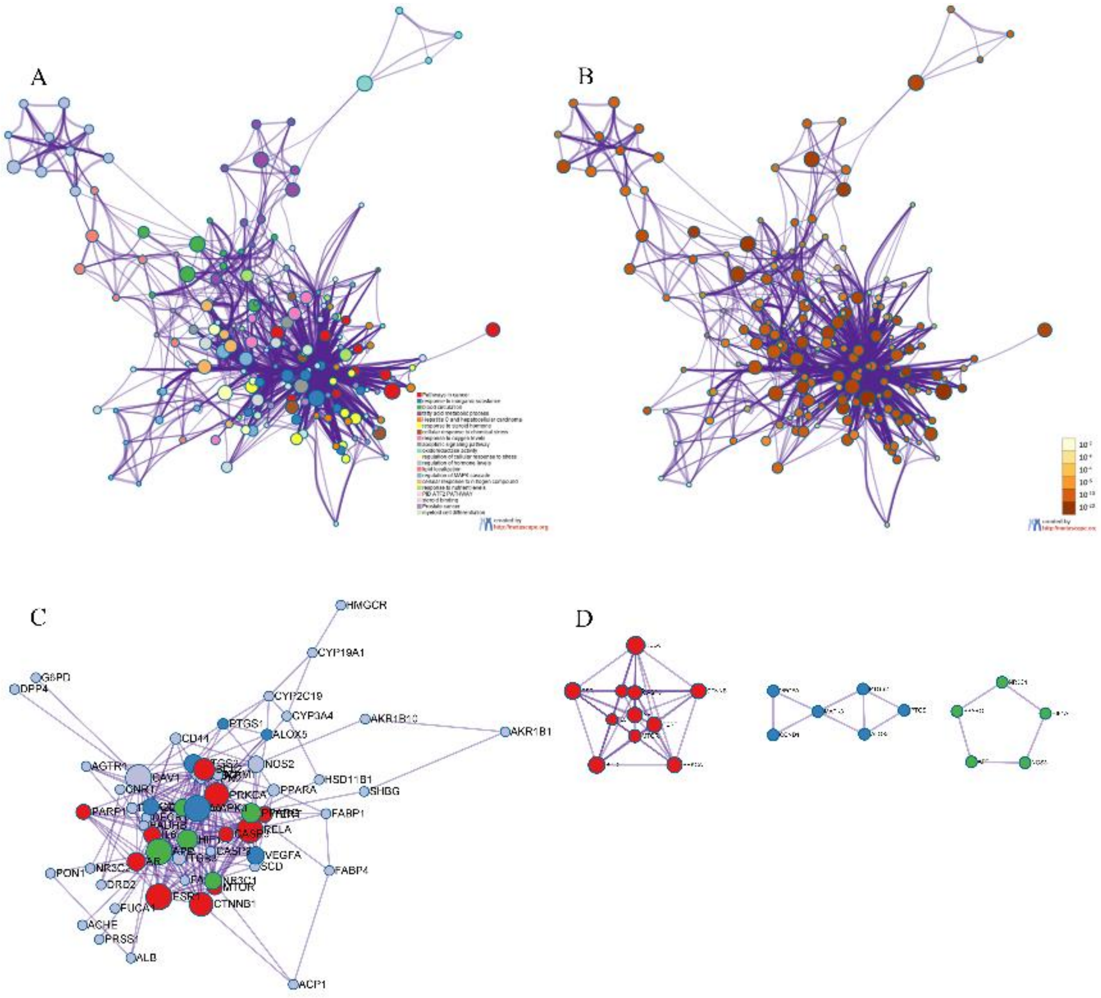

Supplementary figure 1: Network of enriched terms. (A) Colored by cluster ID, nodes that share the same cluster ID are typically close to each other. (B) Colored by p-value, where terms containing more genes tend to have a more significant p-value. (C) Core target screening based on topology analysis. (D) Core target screening based on cluster analysis.

HIF-1α

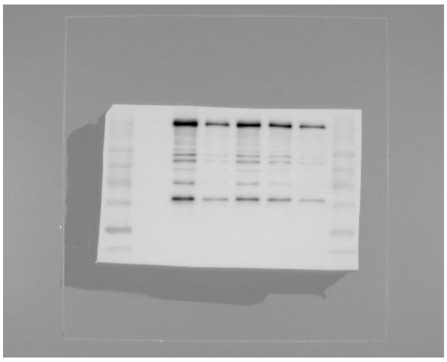

VEGFA

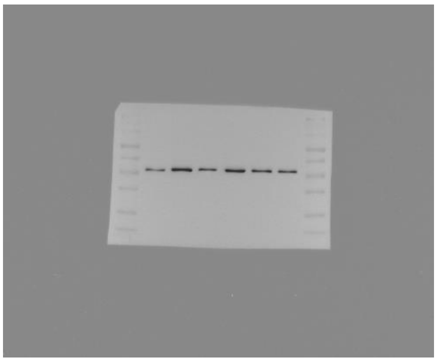

PKC-α

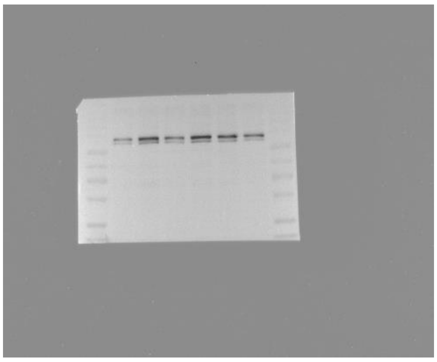

ERK1/2

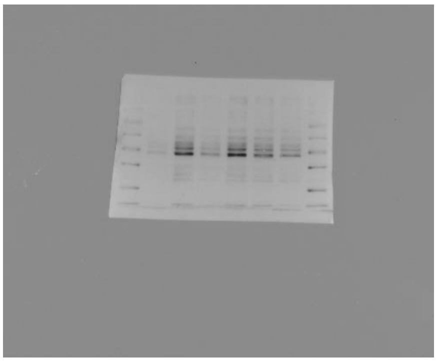

β-tubulin

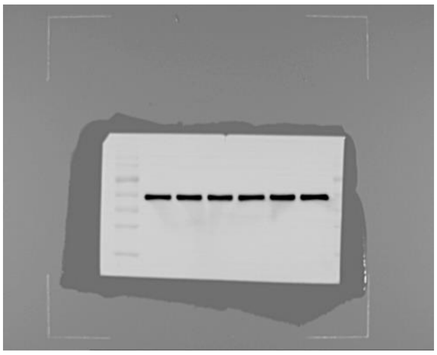

Supplementary figure 2: the full uncropped and unedited versions of the Western blots.
